# The interdependencies of viral load, the innate immune response, and clinical outcome in children presenting to the emergency department with respiratory syncytial virus-associated bronchiolitis

**DOI:** 10.1371/journal.pone.0172953

**Published:** 2017-03-07

**Authors:** Felipe-Andrés Piedra, Minghua Mei, Vasanthi Avadhanula, Reena Mehta, Letisha Aideyan, Roberto P. Garofalo, Pedro A. Piedra

**Affiliations:** 1 Department of Molecular Virology and Microbiology, Baylor College of Medicine, Houston, Texas, United States of America; 2 Allergy & Asthma Specialists, P.C., Saddle River, New Jersey, United States of America; 3 Department of Pediatrics, University of Texas Medical Branch, Galveston, Texas, United States of America; 4 Department of Pediatrics, Baylor College of Medicine, Houston, Texas, United States of America; University of Iowa, UNITED STATES

## Abstract

Respiratory syncytial virus (RSV) causes significant infant morbidity and mortality. For decades severe RSV-induced disease was thought to result from an uncontrolled host response to viral replication, but recent work suggests that a strong innate immune response early in infection is protective. To shed light on host-virus interactions and the viral determinants of disease, copy numbers of five RSV genes (NS1, NS2, N, G, F) were measured by quantitative real-time polymerase chain reaction (qPCR) in nasal wash samples from children with RSV-associated bronchiolitis. Correlations were sought with host cytokines/chemokines and biomarkers. Associations with disposition from the emergency department (hospitalized or sent home) and pulse oximetry O_2_ saturation levels were also sought. Additionally, RNase P copy number was measured and used to normalize nasal wash data. RSV gene copy numbers were found to significantly correlate with both cytokine/chemokine and biomarker levels; and RNase P-normalized viral gene copy numbers (NS1, NS2, N and G) were significantly higher in infants with less severe disease. Moreover, three of the normalized viral gene copy numbers (NS1, NS2, and N) correlated significantly with arterial O_2_ saturation levels. The data support a model where a higher viral load early in infection can promote a robust innate immune response that protects against progression into hypoxic RSV-induced lower respiratory tract illness.

## Introduction

RSV is the most common pathogen associated with lower respiratory tract disease worldwide [1–3]. It causes significant morbidity and mortality among young children, children and adults with co-morbidities, and elderly adults [4, 5].

For decades it was believed that an unrestrained and aberrant host innate immune response was the driver of RSV pathogenesis [6–12]. However, in recent years there has been a shift in thinking as a result of multiple findings reporting protection from severe RSV-induced disease by a robust innate immune response [13–16]. Additionally, numerous host risk factors—such as age, prematurity, and chronic lung disease—and environmental risk factors—such as second hand smoke exposure *in utero* and during infancy, and overcrowding—are well established for severe RSV illness. Less well established are the viral factors and their relationship to disease.

A number of groups, including our own, have investigated the role of viral load in disease with mixed results: some studies report a correlation between viral load and disease severity [17–23], while others report none [24, 25]. Results comparisons are confounded by differences in study design and measurement methods. Most samples have been collected late in infection from hospitalized children [20–23, 25] or from children healthy enough to stay home [19, 24]; and most lack measurements of host response [19–22, 26]. Thus, there is a systematic lack of viral load and host response data, especially from time points early in the course of illness, from young children ultimately experiencing a broad range of disease severity.

RSV gene copy numbers (CNs) provide both a measure of viral load and potential insight into the mechanisms of RSV-induced disease. The nucleocapsid (N) gene encodes the N protein that binds genomic RNA to form a complex which serves as template for viral replication and transcription. We have found the N gene copy number to be directly proportional to the amount of infectious virus *in vitro* (data not shown). The levels of other RSV proteins might depend less strictly on viral load, and could be of particular significance to determining disease severity. The nonstructural 1 and 2 (NS1 and NS2) proteins are not incorporated into virions and inhibit the innate immune response by suppressing induction of type I interferon (IFN) and IFN-inducible genes [27–30]. The attachment (G) protein is one of three surface proteins and is heavily glycosylated. A secretory form of the G protein is believed to act as decoy for neutralizing antibodies [31–33]. The G protein is also able to bind CX3C chemokine receptor 1 (CX3CR1), known as the fractalkine receptor, and inhibit leukocyte chemotaxis [34, 35]. The fusion (F) protein is essential to RSV infectivity and facilitates viral spread by fusing neighboring cells, forming syncytia [36]. F protein also causes toll-like receptor 4 (TLR-4) signaling with NF-κβ and IFN-inducible gene activation early in infection [37]. Thus, transcriptional levels of different genes might differentially affect the innate immune response and disease progression.

We recently showed in a study of children admitted to the ED and diagnosed with bronchiolitis that the induction of a robust innate immune response early in disease resulted in improved clinical outcome [16]. Here, we sought to explore how the copy numbers of five different RSV genes (NS1, NS2, N, G, F) related to host response and disease severity in RSV-infected children from the aforementioned study. We hypothesized a direct relationship between RSV gene copy numbers (CNs) and host cytokine/chemokine responses in the upper respiratory tract with improved clinical outcome. In other words, we hypothesized that a higher viral load early in illness would more effectively engage the innate immune response leading to more rapid resolution of disease. We also explored the host housekeeping gene, RNase P, to normalize both RSV gene CN and host cytokine/chemokine data.

## Results

### Study population

The original study population consisted of 112 children less than 2 years of age with a physician-diagnosis of bronchiolitis made in the ED. Nasal wash samples were collected at presentation to the ED. One or more respiratory viruses were detected in 102 of the 112 children. RSV was the most commonly detected virus and was detected in 79 of the enrolled children—data from these children are reported here. Both RSV/A and RSV/B were detected, and each was represented by one genotype (RSV/A: GA2; RSV/B: BA). Demographic and clinical characteristics of RSV-PCR-positive children at presentation to the ED and by disposition are shown in Table 1. The mean age at presentation was 6.7 months, 60.8% were male, 22.8% were African-American, and 49.4% were Hispanic. Differences in age, gender, race, gestational age at birth, birth weight, examination weight, day care attendance, breast feeding history, tobacco exposure, number of people in house-hold or number of siblings less than 5 years old were not observed between those who were discharged home from the ED versus those who were hospitalized.

**Table 1:** Demographic and clinical characteristics by disposition.

Variables	Disposition			P-values*
	All	Non-Hospitalization	Hospitalization	
	n = 79	n = 38	n = 41	
**Age**				0.76
Mean ±SD	6.7±6.3	6.9±5.7	6.5±6.8	
0–5 months: n (%)	48 (60.8)	22 (57.9)	26 (63.4)	
6 months: n (%)	31 (39.2)	16 (42.1)	15 (36.6)	
**Gender**				0.97
Males: n (%)	48 (60.8)	23 (60.5)	25 (61.0)	
Females: n (%)	31 (39.2)	15 (39.5)	16 (39.0)	
**Race**				0.95
White: n (%)	22 (27.9)	10 (26.3)	12 (29.3)	
Hispanic: n (%)	39 (49.4)	19 (50.0)	20 (48.8)	
African-American: n (%)	18 (22.8)	9 (23.7)	9 (21.9)	
**Gestational age at birth**				
(mean±SD)	38.7±1.3	38.7±1.3	38.6±1.4	0.75
**Birth weight**				0.87
lbs(mean±SD)	7.2±0.9	7.2±0.9	7.2±1.0	
**Examination weight**				0.33
lbs (mean±SD)	16.2±5.7	16.9±5.3	15.4±6.2	
**Day Care**				0.70
Yes: n (%)	20 (25.6)	9 (23.7)	11 (27.5)	
No: n (%)	58 (74.4)	29 (76.3)	29 (72.5)	
**Breastfeeding**				0.95
Yes: n (%)	46 (58.2)	22 (57.9)	24 (58.5)	
No: n (%)	33 (41.8)	16 (42.1)	17 (41.5)	
**Tobacco smoke exposure**				0.83
Yes: n (%)	28 (35.4)	13 (34.2)	15 (36.6)	
No: n (%)	51 (64.6)	25 (65.8)	26 (63.4)	
**No of people at home**				
(mean±SD)	5.0±1.4	4.9±1.3	5.1±1.6	0.51
**No of sibling < 5 yrs old**				
(mean±SD)	1.8±0.8	1.7±0.8	1.9±0.9	0.51
**Duration of illness (days)**				0.005
< 3 days: n (%)	10 (12.7)	7 (18.4)	3 (7.3)	
3–5 days: n (%)	48 (60.7)	16 (42.1)	32 (78.1)	
>5 days: n (%)	21 (26.6)	15 (39.5)	6 (14.6)	
**Respiratory rate**				0.12
Breaths per minute (mean±SD)	49.0±10.9	47.1±10.5	51.4±11.1	
**Heart rate**				0.04
Beats per minute (mean±SD)	161.3±17.3	157.5±17.5	166.1±16.0	
**Body temperature**				0.93
Degrees Fahrenheit (mean±SD)	99.9±1.4	99.9±1.3	100.0±1.6	
**Nasal flaring**				0.0001
Present: n (%)	26 (39.4)	7 (18.9)	19 (65.5)	
Absent: n (%)	40 (60.6)	30 (81.1)	10 (34.5)	
**History of apnea**				0.44
Yes	1 (1.5)	0 (0.00)	1 (3.3)	
No	67 (98.5)	38 (100.0)	3 (96.7)	
**Intercostal retraction**				0.0005
Yes	44 (66.7)	18 (48.7)	26 (89.7)	
No	22 (33.3)	19 (51.3)	3 (10.3)	
**Oxygen saturation at enrollment**				
(mean±SD)	96.5±14.0	99.8±17.4	92.3±5.4	0.02
**IV fluid administration**				<.0001
Given: n (%)	17 (25.0)	2 (5.3)	15 (50.0)	
Not Given: n (%)	51 (75.0)	36 (94.7)	15 (50.0)	
**PO IV steroids**				0.62
Yes: n (%)	14 (20.6)	7 (18.4)	7 (23.3)	
No: n (%)	54 (79.4)	31 (81.6)	23 (76.7)	
**Albuterol or epinephrine administration**				0.21
Yes	40 (50.6)	22 (57.9)	18 (43.9)	
No	39 (49.4)	16 (42.1)	23 (56.1)	
**Antibiotic administration**				0.39
Yes	6 (8.8)	2 (5.3)	4 (13.3)	
No	62 (91.2)	36 (94.7)	26 (86.7)	
**Virus type**				0.21
RSV/A	55 (69.6)	29 (76.3)	26 (63.4)	
RSV/B	24 (30.4)	9 (23.7)	15 (36.6)	
**Virus infection pattern**				0.99
Single	52 (65.8)	25 (65.8)	27 (65.9)	
co-infection	27 (34.2)	13 (34.2)	14 (34.2)	

* Chi-square tests or Fisher’s exact tests for categorical variables and t-tests for continuous variables

No differences were observed in the pattern of virus infection (single or co-infected) or virus type (RSV/A or RSV/B) between non-hospitalized and hospitalized children. Duration of illness (DOI) at presentation to the ED was significantly different between the non-hospitalized and hospitalized groups (P = 0.005). Most children with hospitalized bronchiolitis presented between days 3 to 5 of illness onset (78.1%), while children with non-hospitalized bronchiolitis were more evenly distributed at presentation to the ED.

Children with bronchiolitis who were hospitalized experienced more severe disease as manifested by a significantly greater proportion with nasal flaring, intercostal retraction, lower oxygen saturation at enrollment, and need for intravenous fluids. There were no differences between the two groups in the administration of albuterol or epinephrine, oral or intravenous steroids or antibiotics.

### RSV copy numbers

Copy numbers (CNs) of 5 different RSV genes (NS1, NS2, N, G, and F) were measured in nasal wash (NW) samples from infants diagnosed with bronchiolitis and PCR-positive for RSV. RNase P gene CN was also measured in each sample and used to normalize RSV gene CNs to control for variable sample quality.

Viral gene CNs showed high variability (S1 Table) but all were significantly different from each other by Wilcoxon signed-rank test. Of the 5 viral genes measured, mean G CN was highest and mean NS2 CN was lowest (S1 Table). Relative gene expression levels remained unchanged after RSV CNs were normalized with RNase P (Table 2).

**Table 2:** Description of the RNase P-normalized copy numbers of 5 RSV genes.

		All			RSV/A			RSV/B	
	N	Mean(median)	SD	N	Mean(median)	SD	N	Mean (median)	SD
NS1	79	77.1 (18.4)	141.7	55	91.4 (22.1)	163.8	24	44.3 (16.3)	59.2
NS2	79	17.2 (3.6)	32.6	55	15.6 (3.6)	28.0	24	21.0 (3.6)	41.8
N	79	76.1 (17.1)	136.0	55	80.0 (16.8)	152	24	67.1 (22.7)	91.5
G	79	110.6 (30.0)	173.3	55	128.6 (34.9)	197.1	24	69.3 (23.1)	89.7
F	79	47.1 (7.9)	86.2	55	51.3 (5.5)	98.7	24	37.3 (10.8)	46.9

Note: RNase P-normalized RSV gene copy number (CN) = RSV gene CN/RNase P CN.

**Fig 1:**
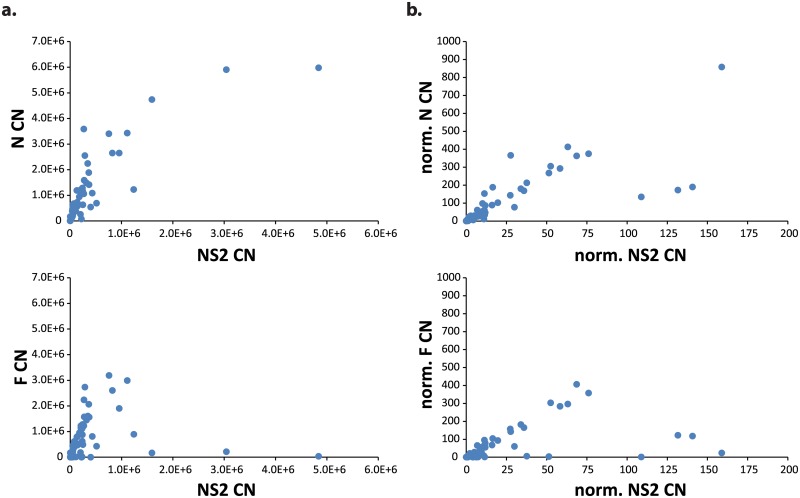
RSV gene Copy Numbers (CN) are correlated highly and linearly, and correlations improve when data are normalized by RNase P CN. (A) N CN vs. NS2 CN showing the most correlated pair of CNs measured (r = 0.941; p<0.0001); F CN vs. NS2 CN showing the least correlated pair of CNs measured (r = 0.789; p<0.0001). (B) Normalized N CN vs. normalized NS2 CN (where normalized CN = RSV gene CN/ RNase P CN) showing the most correlated pair of normalized CNs measured (r = 0.963; p<0.0001); normalized F CN vs. normalized NS2 CN showing the least correlated pair of normalized CNs measured (r = 0.808; p<0.0001).

CN differences between RSV/A- and RSV/B-infected children were not significant by Wilcoxon-Mann-Whitney tests and thus for subsequent analyses the gene CNs for RSV/A and RSV/B infections were combined. The CNs of all 5 RSV genes correlated highly with each other (S2 Table)(Table 3). Correlation strengths increased for all CN pairs but F and NS1 after normalizing by RNase P CN. N and NS2 were the most correlated pair of CNs measured, while F and NS2 were the least correlated (Table 3)(Fig 1). Additionally, no differences were observed in RSV gene CNs when grouped by duration of illness (DOI) (< 3, 3–5, & >5 days) using Wilcoxon-Mann-Whitney tests.

**Table 3:** Spearman correlations among the RNase P-normalized copy numbers of 5 RSV genes.

	N	**NS1**	**NS2**	**N**	**G**	**F**
**NS1**	79	-	0.917 (<.0001)	0.960 (<.0001)	0.946 (<.0001)	0.869 (<.0001)
**NS2**	79		-	0.963 (<.0001)	0.930 (<.0001)	0.808 (<.0001)
**N**	79			-	0.948 (<.0001)	0.863 (<.0001)
**G**	79				-	0.831 (<.0001)
**F**	79					-

Note: RNase P-normalized RSV gene copy number (CN) = RSV gene CN/RNase P CN.

### Correlations with cytokine & bronchiolitis biomarker levels

We previously demonstrated that higher levels of cytokines/chemokines (IFN-gamma, IL-4, IL-15, IL-17, IP-10, and eotaxin) were significantly associated with a decreased risk of hospitalization [16]. These cytokines/chemokines have major roles in Th1 (IFN-gamma), Th2 (Il-4), regulatory (Il-17), maturational (Il-15), and chemoattractant (IP-10 and eotaxin) processes of the innate and adaptive immune responses. Correlations between these clinically significant host cytokine/chemokine levels and RSV gene CNs were sought to explore our data for relationships between viral load/gene expression and host immune response. RSV gene CNs strongly correlated with levels of IP-10 (also known as CXCL10), showing no correlation with the other cytokines measured (IFN-gamma, IL-4, IL-15, IL-17, eotaxin) (S3 Table); however, after normalizing RSV gene CNs and host cytokine/chemokine levels by RNase P, significant correlations were observed between RSV gene CNs and the remaining cytokines/chemokines measured (IFN-gamma, IL-4, IL-15, IL-17, and eotaxin) (Table 4). Correlations with IP-10 remained essentially unchanged after normalization with RNase P. It is important to note that cytokine levels were not affected by the RSV infection pattern (single vs. co-infection). Infants with RSV as the sole pathogen had cytokine levels comparable to infants with RSV co-infection (data not presented).

**Table 4:** Spearman correlations between RNase P-normalized RSV gene copy numbers and cytokine levels.

	N	IFN-γ	IL-4	IL-15	IL-17	Eotaxin	IP-10
**NS1**	68	0.43(0.0003)	0.28(0.019)	0.39(0.0009)	0.29(0.01)	0.33(0.006)	0.54 (<.0001)
**NS2**	68	0.39(0.001)	0.24(0.05)	0.36(0.0025)	0.26(0.03)	0.32(0.008)	0.51 (<.0001)
**N**	68	0.46 (<.0001)	0.28(0.02)	0.43(0.0003)	0.30(0.01)	0.37(0.0017)	0.59 (<.0001)
**G**	68	0.37(0.002)	0.26(0.03)	0.34(0.004)	0.25(0.04)	0.29(0.01)	0.48 (<.0001)
**F**	68	0.35(0.0035)	0.20(0.09)	0.30(0.01)	0.25(0.04)	0.25(0.04)	0.45(0.0001)

Note: RNase P-normalized RSV gene copy number (CN) = RSV gene CN/RNase P CN; RNase P-normalized [cytokine] = [cytokine] (pg/ml)/RNase P CN.

To further explore the host innate immune response to varying RSV gene CNs, correlations with additional cytokines from multiple major functional groups were sought. Based on normalized data, RSV gene CNs correlated highly with 1) most cytokines measured in Th2 and regulatory groups; 2) most chemoattractant cytokines; and 3) a number of maturational and Th1 cytokines (S3 Table). Normalized RSV gene CNs showed no correlation with all but one pro-inflammatory cytokine (S3 Table). These data are consistent with a broad cytokine-mediated host innate immune response to RSV, one with magnitude that depends on viral load.

RSV gene CNs were also analyzed for correlations with levels of various bronchiolitis biomarkers. Lactate dehydrogenase (LDH) and caspase were measured as described earlier [15] along with the activity levels of myeloperoxidase (MPO) and matrix metalloproteinase-7 (MMP-7). LDH activity levels are a measure of cellular injury, and caspase activity reports on apoptosis. MPO is a marker of polymorphonuclear cell degranulation, and MMP-7 is an endopeptidase that breaks down extracellular matrix proteins. All RSV gene CNs correlated with caspase activity levels, suggesting a pro-apoptotic effect of RSV infection in our patient cohort; and most RSV gene CNs correlated with activity levels of LDH, MPO, and MMP-7 (S4 Table). Normalizing both RSV gene CNs and biomarker activity levels by RNase P CN improved correlation coefficients of most paired comparisons (Table 5).

**Table 5:** Spearman correlations between RNase P-normalized RSV gene copy numbers and biomarker activity levels.

	N	NW LDH	NW Caspase	NW MMP7	NW MPO
**NS1**	79	0.35(0.002)	0.49 (<.0001)	0.27(0.01)	0.42(0.0001)
**NS2**	79	0.32(0.004)	0.48 (<.0001)	0.28(0.01)	0.42(0.0001)
**N**	79	0.39(0.0004)	0.55 (<.0001)	0.33(0.003)	0.50 (<.0001)
**G**	79	0.36(0.001)	0.50 (<.0001)	0.33(0.003)	0.41(0.0002)
**F**	79	0.29(0.009)	0.43 (<.0001)	0.26(0.02)	0.37(0.0008)

Abbreviations: LDH = Lactate dehydrogenase; MPO = myeloperoxidase; MMP-7 = matrix metalloproteinase-7 (MMP-7).

Note: RNase P-normalized RSV gene copy number (CN) = RSV gene CN/RNase P CN; RNase P-normalized [biomarker] = [biomarker] (μU/ml)/RNase P CN.

Significant correlations were observed between RSV gene CNs and host cytokine/chemokine and biomarker levels. To better understand the connections between RSV gene copy numbers and host factors, a multivariate logistic regression model with backward elimination was used. Separate models were made for RNase P-normalized NS1, NS2, N, G and F gene CNs. Normalized RSV gene CN was analyzed by quartiles and made into a categorical outcome variable [Q4 (highest quartile) versus combined Q1, Q2 & Q3 (reference)]; the independent variables included in the model were age (<6 & ≥6 months), DOI at enrollment (<3 days & ≥3 days), disposition (hospitalized, sent home), RSV subgroup (RSV/A & RSV/B) and normalized cytokines/biomarkers (IL-17, IP-10, LDH, and MPO). IP-10 associated with RSV gene CN in 4 of the 5 models. Odds ratios (OR) for IP-10 were as follows: 1) NS1 model: OR = 1.27 (95% CI: 1.10, 1.46); 2) NS2 model: OR = 1.31 (95% CI: 1.12, 1.52); 3); N model: OR = 1.33 (95% CI: 1.14, 1.56); and 4) G model: OR = 1.11 (95% CI: 1.03, 1.20). None of the independent variables used in the F model was found to be significant. Similar results were obtained when neither the RSV gene CNs nor the cytokines/biomarkers were normalized by RNase P, with IP-10 alone associating significantly with RSV gene CN in 4 of the 5 models.

### Associations with clinical outcome

RSV gene CNs were analyzed for differences between infants who were hospitalized (ACU or PICU) and infants who were sent home after presenting in respiratory distress to the ED. Interestingly, NS1 CN (S5 Table) and all normalized RSV gene CNs but F were significantly higher in infants who were not hospitalized (Table 6). RNase P-normalized NS1, NS2, N, and G gene CNs in the non-hospitalized group exceeded those in hospitalized children by approximately 4- to 6-fold.

**Table 6:** RNase P-normalized RSV gene copy numbers between non-hospitalized and hospitalized infants.

	Non-hospitalized		Hospitalized		
	N	Median (Q1-Q3)	N	Median (Q1-Q3)	P-value
**NS1**	38	47.25 (5.62–108.13)	41	8.26 (0.56–29.77)	0.01
**NS2**	38	8.07 (1.49–16.48)	41	1.61 (0.37–6.87)	0.02
**N**	38	32.72(6.04–168.91)	41	8.85(1.49–29.57)	0.03
**G**	38	70.01(9.98–182.36)	41	14.55(2.57–97.10)	0.04
**F**	38	16.78 (0.82–78.77)	41	6.02(0.30–17.04)	0.21

Differences between groups were evaluated by Wilcoxon-Mann-Whitney tests. P value <0.05 was considered significant.

Note: RNase P-normalized RSV gene copy number (CN) = RSV gene CN/RNase P CN.

To better understand the determinants of disease severity in our patient cohort, multivariate logistic regression analyses were performed using disposition (hospitalized or not) as outcome. Five models were generated with backward elimination. Each model included a different normalized RSV gene CN (Q4 & combined Q1, Q2, Q3), selected normalized cytokines and biomarkers (IP-10, IL-17, LDH, & MPO), age (<6 & >6 months), DOI at enrollment (<3 days & ≥3 days), and RSV subgroup (RSV/A or RSV/B). In all 5 models IP-10 was identified as an independent factor influencing disposition, with higher levels predicting less severe disease (all odds ratios (OR) = 0.85 (95% CI: 0.75, 0.96)). LDH was also significantly associated with disposition, with higher levels predicting more severe disease (all OR = 1.03 (95% CI: 1.01, 1.06)). Similar results were obtained when neither the RSV gene CNs nor the cytokines/biomarkers were normalized by RNase P, with higher levels of IP-10 predicting less severe disease (all OR = 0.12; 95% CI: 0.02, 0.76) and higher levels of LDH predicting more severe disease (all OR = 7.0 (95% CI: 1.5, 31.8)) in all 5 models. Un-normalized IL-17 also associated with disposition in these analyses, with higher levels, like IP-10, predicting improved clinical outcome (all OR = 0.34; 95% CI: 0.16, 0.75).

Because disposition (hospitalized or not) is binary and potentially influenced by subjective criteria, O_2_ saturation levels measured by pulse oximetry were used as an objective measure of airway injury and/or obstruction in children with bronchiolitis. Correlations were determined between O_2_ saturation levels and 1) RSV gene CNs, 2) cytokines/chemokines (IFN-gamma, IL-4, IL-15, IL-17, IP-10, and eotaxin), and 3) biomarkers (LDH, caspase, MPO, and MMP-7). Significant correlations were observed for normalized RSV gene CNs NS1, NS2 and N (all P = 0.03 and r = 0.28); the cytokines IFN-gamma (P = 0.04, r = 0.26) and Il-15 (P = 0.24, r = 0.26); and a significant inverse correlation was observed for LDH (P = 0.03, r = -0.29).

## Discussion

To help unravel the contributions of viral cytopathic effect and host immune response to RSV pathogenesis, RSV gene copy numbers (CNs) were measured and analyzed for correlations with cytokine/chemokine levels, biomarker levels, and clinical outcome in samples collected from RSV-infected infants admitted to the ED.

RSV gene CNs correlated with a diversity of cytokines and all biomarkers measured. RSV gene CNs were higher in infants with less severe disease (using RNase P-normalized data); and significant correlations were found between normalized RSV gene CNs (NS1, NS2, N) and arterial O_2_ saturation levels. Although potentially counter-intuitive, we believe that we can rationalize the apparent disease-mitigating effect of higher viral loads observed in our study.

### More RSV early in infection, less severe disease

Our data come from samples collected shortly after patients were admitted to the ED and therefore represent earlier time-points with respect to the start of infection than data from most comparable studies [17–23]; and because these are single time-point data, we need only understand how more virus early in infection (not throughout) can lead to less severe disease. Furthermore, although RSV gene CNs were significantly higher among infants who were not hospitalized, we did not observe an association between any of the 5 RSV gene CNs and disposition (hospitalized or not) by multivariate logistic regression; however, we did observe a strong association between RSV gene CNs and levels of IP-10, and the latter associated strongly with disposition and predicted an improved outcome. A potential role of IP-10 is to induce a protective T cell response that improves clearance of infected respiratory epithelial cells through promotion of dendritic cell maturation, T cell stimulation, and IL-12 secretion [38]. Thus, higher RSV gene CNs might only protect through the extent to which they elicit a potent host response early in infection. Stated differently, higher RSV gene CNs early in infection might bias the host toward more mild disease through increased levels of directly protective cytokines. A higher multiplicity of infection (MOI) could therefore work against viral replication by enabling early detection of virus and thereby favoring a more rapid and productive host response. We have observed a protective effect from viral load (measured by pfu) in a similar population (P.A.P. personal communication) [13]. We therefore suspect that the time-scale of the host innate immune response to infection is highly important to determining RSV disease severity, where the swifter and more robust the response (rapid increases in array of cytokines) the less likely severe disease is to ensue.

Higher MOIs might also be directly protective. Sun *et al*. showed that defective viral genomes (DVGs) of RSV promote a strong innate antiviral response in mice and humans [39]. DVGs are noninfectious viral genomes and particles that result from errors in viral replication [40]. They are commonly observed in cell culture at high MOIs [41]. Higher MOIs might favor the generation of DVGs by increasing the likelihood of nonspecific interactions between viral proteins that lead to ‘errors’ during replication. Critically, some DVGs (copy- and snap-back DVGs) are completely antagonistic to viral replication because their genes cannot be transcribed due to the absence of a 3’ leader sequence where viral polymerases load [40]. Any DVGs present in our samples would have contributed to our CN measurements.

Contrary to what we report here, a number of studies including our own have reported an association between higher viral load and greater disease severity [17–23]. Recently we found a higher risk of intensive care in children with higher RSV genomic load [17]. In the latter study, sample quality was not measured and therefore could not be corrected by RNase P copy number. Additionally, samples were collected from hospitalized infants only and later into the course of infection compared to the current study [17] Others have reported similar associations between viral load and disease severity, but the associations are weak and come either from hospitalized infants [20–23] or infants healthy enough to stay home [19, 24]–rarely both [26]. Additionally, most of the samples were collected later in the course of infection than those analyzed here. Among other things, the single time-point nature of these studies makes discovering the determinants of disease severity difficult. DeVincenzo *et al*. performed a longitudinal study by infecting healthy adult volunteers with variable amounts of RSV and measuring their symptoms and viral load through time [18]. They concluded that viral load drove disease severity in their study yet made clear that measured viral loads also correlated with levels of IL-6 and IL-8, and were independent of the inoculating dose [18]. What host factors determined variable viral loads and disease severity in their study is entirely unclear; thus, even in healthy adults what drives the severity of RSV-induced disease remains unknown.

### RSV gene CNs correlate with each other, cytokine/chemokine and biomarker levels

All RSV gene CNs (NS1, NS2, N, G, F) correlated strongly and linearly with each other, and all were significantly different. CNs reflect the amount of 1) genomic and anti-genomic RNA (whole and, potentially, defective), and2) mRNA present in our samples. The correlations and differences observed are perhaps not surprising as all RSV gene CNs will depend on viral load, leading to correlations; each RSV gene CN will also depend on a potentially different rate of transcription and transcript stability, leading to differences. Most correlations improved somewhat after correcting for sample quality by RNase P CN.

RSV gene CNs correlated with levels of numerous cytokines from an array of functional groups and the activity levels of established bronchiolitis biomarkers (caspase and LDH). Although increases in both are an expected part of host resistance to viral replication, we did not expect to observe an apparent RSV gene CN-dependent response. Consistent with previous findings (P.A.P. personal communication), these results suggest that the magnitude of the host response to infection with RSV depends on viral load.

Both cytokine and biomarker levels were correlated with RSV gene CNs yet gave qualitatively different odds ratios in multivariate analyses with disposition as outcome (protective and not, respectively). This is reasonable assuming increases in both are temporally distinct, as a simultaneous increase would suggest a contradictory host response. Additionally, it is misleading to call one protective and the other not, because both contribute to or reflect host resistance to viral replication—with the partial exception of LDH, whose levels reflect both apoptosis and/or necrosis of host tissue. Furthermore, we have reported associations in similar cohorts between bronchiolitis biomarker levels and both more and less severe disease [15, 42]. Systematic differences in when samples were collected during the course of infection might account for these differing results. Nevertheless, increased activity levels of caspase and LDH are clearly associated with RSV-induced disease, but not necessarily predictive of disease severity.

### Study limitations

This study did not have an uninfected control group for measurements of and analyses including cytokine levels. Although the absence of an uninfected control group does not alter our RSV gene CN findings in relation to clinical outcomes. Samples were collected from infants early in the course of infection, but it would be ideal to have multi-time-point data spanning its full duration; dynamics measurements of 1) RSV gene CNs and 2) host cytokine/chemokine and biomarker levels coupled to a range of clinical outcomes might help reveal the determinants of severe disease. Additionally, no measurements were made to test for the presence of DVGs in our samples. Testing for DVGs is important for future studies; however, the major hypothesis to emerge from our data does not depend on their presence.

Our results support the hypothesis that more virus early in illness can lead to less severe RSV-induced disease. This might result from higher viral loads—potentially through the increased generation of DVGs—inducing a swifter host immune response early in infection. We speculate that a delayed and/or weak host response early in infection is more likely to lead to prolonged viral replication with subsequent tissue injury reflecting clinically as severe disease.

## Materials and methods

### Study design

This was a cross-sectional, prospective, single-site study conducted at Texas Children’s Hospital from October 2010 through April 2011 during the bronchiolitis season in Houston, TX [13, 15]. Findings from this study have been previously reported [13, 15]. Healthy children less than 24 months of age without a known co-morbid condition who presented to the ED with respiratory distress and were diagnosed with bronchiolitis by their attending physician were eligible for study participation [13]. The ED supervising physician made the evaluation and final disposition of the child without any input from the research team. Bronchiolitis was defined as a physician diagnosis in children less than 24 months of age with wheezing and/or rales who had a history of preceding upper airway illness. After enrollment in the ED, a single nasal pharyngeal aspirate was collected in the ED and transported in viral transport media to the Respiratory Virus Diagnostic Laboratory (CLIA ID 45D0919666) at Baylor College of Medicine. Viral testing was performed via cell culture and real-time PCR for RSV (A and B), and other respiratory viruses [13, 15]. Demographic and clinical data were obtained at enrollment and extracted from the medical record. The caregivers were called 7 to 14 days after discharge from the ED or hospital to ensure there was no change in disposition. A child participated in the study only after obtaining written informed consent from a parent or legal guardian, and met all the inclusion criteria and none of the exclusion criteria. The inclusion criteria were the child was previously healthy and had a physician diagnosis of bronchiolitis in the ED. The exclusion criteria were co-morbid medical conditions such as chronic lung disease, cyanotic congenital heart disease, neuromuscular disease, a primary immunodeficiency, prematurity (<36 weeks) or had respiratory distress unrelated to a viral URI. This study was approved by the Institutional Review Board (IRB) of Human Subject Research at Baylor College of Medicine and Affiliated Institutions which includes Texas’s Children Hospital. The IRB is registered with the Office for Human Research Protections of the U.S. Department of Health and Human Services.

### RNA extraction, reverse transcription and genotyping

Viral RNA was extracted from NW samples as described [43] by using the Mini Viral RNA Kit (Qiagen Sciences, Germantown, Maryland) and automated platform QIAcube (Qiagen, Hilden, Germany) according to the manufacturer instructions. Complementary DNA (cDNA) was generated by superscript VILO according to the manufacturer’s instructions (Life Technologies, Applied Biosystems, Carlsbad, California). Samples were genotyped as described [44, 45] by sequencing a 270 bp fragment in the 2^nd^ hypervariable region of the G gene.

#### Measurements of RSV and RNase P copy numbers

Subgroup-specific primers and probes were generated for quantitative real-time polymerase chain reaction (qPCR) -based measurements of copy numbers of 5 different RSV genes: NS1, NS2, N, G, and F. Primers and probes were designed using sequences available from Genbank: U50362 (RSV A2) and NC_001781 (RSV B1). Reaction conditions were optimized to achieve amplification efficiencies greater than or equal to 90%.

To measure RSV gene copy numbers, the aforementioned sequences (U50362 and NC_001781) were used to generate subgroup-specific oligonucleotide standards. Standards were purchased from IDT^®^, received lyophilized and resuspended in nuclease-free water. For each oligonucleotide standard a concentration was found at which the cycle threshold value (*C*_T_) measured by qPCR was at or close to 15 (around 1.0 pg/μl), with thresholding done according to the manufacturer’s instructions [46]. For each standard, a series of 10-fold dilutions was made starting from the aforesaid concentration. *C*_T_s were then measured for: 1) each series of 10-fold standard dilutions; and 2) the appropriate cDNAs acquired from clinical samples. Samples were measured in duplicate, and copy numbers were determined by mapping unknown sample *C*_T_s to the linear relationship measured for standard *C*_T_ vs. log10 of the standard concentration.

RNase P primers and probes were acquired from the Centers for Disease Control and Prevention (CDC). RNase P was measured in a number of human cell lines (Hep-2, A549, Calu-3). Briefly, HEp-2, A549, and Calu-3 cells were separately grown and harvested, counted with a hemocytometer, lysed by sonication and pelleted; RNAs in the supernatant were extracted and converted to cDNA. RNase P copy numbers were measured using standard dilutions of HEp-2 cells at known concentration. Thus, a sample’s RNase P copy number is equal to the number of HEp-2 cells needed to measure the same amount of RNase P. So long as the amplification efficiency remains constant, oligonucleotide standards or any other human cell line would suffice for generating RNase P copy numbers needed to adjust data for sample quality.

### Cytokine & biomarker quantification

Cytokine levels come from those reported in Nicholson *et al*. [16] and were measured as described by use of a Bio-Plex Human Cytokine Assay (Bio-Rad Laboratories) including interleukin (IL)-1B, IL-1ra, IL-2, IL-4, IL-5, IL-6, IL-7, IL-8, IL-9, IL-10, IL-12, IL-13, IL-15, IL-17, granulocyte colony-stimulating factor (G-CSF), granulocyte-macrophage colony-stimulating factor (GM-CSF), interferon (IFN)-γ, monocyte chemoattractant protein (MCP)-1, macrophage inflammatory protein (MIP)-1a, MIP-1b, vascular endothelial growth factor (VEGF), platelet-derived growth factor (PDGF)-bb, basic fibroblast growth factor (bFGF), chemokine (C-C motif) ligand (CCL) 2, CCL3, CCL4, CCL5, C-X-C motif chemokine 10 (also known as IP-10), eotaxin and tumor necrosis factor (TNF)-α.

Caspase and LDH levels were measured as described [15] using the Caspase-Glo 3/7 Assay (Promega, Madison, WI, USA) and Cytotoxicity Detection Kit (Roche Applied Science, Indianapolis, IN, USA), respectively [15]. MMP-7 and MPO levels were measured using Human Total MMP-7 and Human Myeloperoxidase Quantikine ELISA Kits (R&D systems Minneapolis, MN, USA).

### Statistical analysis

Copy numbers (CNs) measured by qPCR of 5 RSV genes (NS1, NS2, N, G and F) were either left alone (raw) or normalized by RNase P copy number for comparisons. To evaluate our data for relationships between RSV gene CNs and other molecular factors (cytokine/chemokine and biomarker levels) correlations were calculated using Spearman’s coefficient. For descriptive statistics, continuous variables were represented as mean and standard deviation (SD) or median (IQR (Q3-Q1)); categorical variables were represented as frequencies or corresponding percentages. Further analyses were conducted to test for association between RSV gene CNs and clinical outcome. Patients were separated into 2 groups for the primary outcome disposition: those who were hospitalized (children cared for in the acute care unit (ACU) or the pediatric intensive care unit (PICU)) and those who were not hospitalized (children discharged from the ED or observed for <24 hrs in the observation unit before being discharged home). Participants’ ages were divided into two groups: 0–5 months and ≥6 months. Duration of illness (DOI) at presentation was separated into three groups: 0–2 days, 3–5 days and >5 days or two groups: 0–2 and ≥3 days. Patients selected were exclusively infected with either RSV/A or /B. Wilcoxon-Mann-Whitney tests were used to test for differences of raw and normalized RSV gene CNs by disposition (hospitalized vs. not hospitalized) and DOI (0–2 days and ≥3 days). For the identification of independent factors (including RSV gene CNs and cytokine levels) that may influence disposition (hospitalized vs. not hospitalized), multivariable logistic regression analyses were performed to calculate odds ratios (OR) and corresponding 95% confidence intervals (95% CI). Final models were established based on multivariable logistic regression analyses with backward elimination. To determine the predictors that modified RSV gene CNs, using the categorized copy numbers (two groups: top quartile (Q4) vs. lower 3 quartiles combined (Q1, Q2 and Q3)) as dependent variables, multivariate models were also developed while controlling for age, DOI, disposition of patients and virus infection type and cytokines. Similar multivariate analyses were performed using disposition (hospitalized or not) as dependent variable. All statistical analyses were performed using the SAS software package version 9.4 (SAS Institute, Inc., Cary, NC, USA).

## Supporting information

S1 Table Description of the raw copy numbers of 5 RSV genes and RNase P (endogenous control). (DOCX)

S2 Table Spearman correlations among the raw copy numbers of 5 RSV genes. (DOCX)

S3 Table Spearman correlations between RSV gene copy numbers and the levels of a diversity of cytokines. (DOCX)

S4 Table Spearman correlations between raw RSV gene copy numbers and biomarker activity levels. (DOCX)

S5 Table Raw RSV gene copy numbers between non-hospitalized and hospitalized infants. (DOCX)
