# Supplementary material for: The interdependencies of viral load, the innate immune response, and clinical outcome in children presenting to the emergency department with respiratory syncytial virus-associated bronchiolitis
Source: PLoS One. 2017 Mar 7;12(3):e0172953. doi: 10.1371/journal.pone.0172953 (PMC5340370; doi:10.1371/journal.pone.0172953)
Supplement: S1 Table — (DOCX) [file pone.0172953.s001.docx]

**S1 Table. Description of the raw copy numbers of 5 RSV genes and RNase P (endogenous control).**

|  |  | **All** |  |  |  | **RSV/A** |  |  |  | **RSV/B** |  |
| --- | --- | --- | --- | --- | --- | --- | --- | --- | --- | --- | --- |
|  | **N** | **Mean(median)** | **SD** |  | **N** | **Mean(median)** | **SD** |  | **N** | **Mean (median)** | **SD** |
| Raw copy numbers of 5 RSV genes^1^ |  |  |  |  |  |  |  |  |  |  |  |
| NS1 | 79 | 111.6 (36.7) | 230.1 |  | 55 | 138.1 (37.3) | 267.6 |  | 24 | 51.1 (27.8) | 77.5 |
| NS2 | 79 | 28.5 (7.5) | 67.8 |  | 55 | 23.8 (7.6) | 47.7 |  | 24 | 39.3 (6.5) | 100.3 |
| N | 79 | 88.8 (40.9) | 126.8 |  | 55 | 84.5 (35.1) | 118.3 |  | 24 | 98.5 (55.4) | 146.8 |
| G | 79 | 216.6 (72.0) | 466.2 |  | 55 | 263.2 (85.6) | 542.9 |  | 24 | 109.9 (56.6) | 167.6 |
| F | 79 | 56.1 (18.6) | 77.8 |  | 55 | 56.5 (17.2) | 74.8 |  | 24 | 55.2 (22.7) | 86.0 |
| Endogenous control |  |  |  |  |  |  |  |  |  |  |  |
| RNase P^1^ | 79 | 9.0 (1.3) | 18.3 |  | 55 | 10.9 (1.2) | 21.2 |  | 24 | 4.4 (2.0) | 6.5 |

^1^Copy numbers in table = actual mean, median, or SD/10^4^.
