# Supplementary material for: The interdependencies of viral load, the innate immune response, and clinical outcome in children presenting to the emergency department with respiratory syncytial virus-associated bronchiolitis
Source: PLoS One. 2017 Mar 7;12(3):e0172953. doi: 10.1371/journal.pone.0172953 (PMC5340370; doi:10.1371/journal.pone.0172953)
Supplement: S2 Table — (DOCX) [file pone.0172953.s002.docx]

**S2 Table. Spearman correlations among the raw copy numbers of 5 RSV genes.**

|  | **N** | **Un-normalized** | | | | |
| --- | --- | --- | --- | --- | --- | --- |
| **Un-normalized** |  | **NS1** | **NS2** | **N** | **G** | **F** |
| **NS1** | 79 | - | 0.850(<.0001) | 0.917(<.0001) | 0.906(<.0001) | 0.883(<.0001) |
| **NS2** | 79 |  | - | 0.941(<.0001) | 0.889(<.0001) | 0.789(<.0001) |
| **N** | 79 |  |  | - | 0.932(<.0001) | 0.845(<.0001) |
| **G** | 79 |  |  |  | - | 0.833(<.0001) |
| **F** | 79 |  |  |  |  | - |
