# Supplementary material for: The interdependencies of viral load, the innate immune response, and clinical outcome in children presenting to the emergency department with respiratory syncytial virus-associated bronchiolitis
Source: PLoS One. 2017 Mar 7;12(3):e0172953. doi: 10.1371/journal.pone.0172953 (PMC5340370; doi:10.1371/journal.pone.0172953)
Supplement: S3 Table — (DOCX) [file pone.0172953.s003.docx]

**S3a Table. Spearman correlations between RSV gene copy numbers and the levels of 6 cytokines.**

|  |  | **Un-normalized cytokines** | | | | | |
| --- | --- | --- | --- | --- | --- | --- | --- |
| **Un-normalized copy numbers** | **N** | **IFN-γ** | **IL-4** | **IL-15** | **IL-17** | **Eotaxin** | **IP-10** |
| **NS1** | 68 | 0.23(0.06) | 0.19(0.11) | 0.10(0.42) | 0.14(0.24) | 0.04(0.73) | 0.53(<.0001) |
| **NS2** | 68 | 0.14(0.25) | 0.14(0.25) | 0.07(0.58) | 0.11(0.39) | 0.07(0.57) | 0.48(<.0001) |
| **N** | 68 | 0.21(0.10) | 0.15(0.21) | 0.07(0.59) | 0.10(0.42) | 0.05(0.68) | 0.54(<.0001) |
| **G** | 68 | 0.16(0.19) | 0.16(0.21) | 0.08(0.54) | 0.09(0.45) | 0.09(0.47) | 0.47(<.0001) |
| **F** | 68 | 0.19(0.13) | 0.12(0.31) | 0.09(0.44) | 0.13(0.32) | 0.03(0.78) | 0.47(<.0001) |
|  |  | **Normalized cytokines** | | | | | |
| **Normalized copy numbers** |  | **IFN-γ** | **IL-4** | **IL-15** | **IL-17** | **Eotaxin** | **IP-10** |
| **NS1** | 68 | 0.43(0.0003) | 0.28(0.019) | 0.39(0.0009) | 0.29(0.01) | 0.33(0.006) | 0.54(<.0001) |
| **NS2** | 68 | 0.39(0.001) | 0.24(0.05) | 0.36(0.0025) | 0.26(0.03) | 0.32(0.008) | 0.51(<.0001) |
| **N** | 68 | 0.46(<.0001) | 0.28(0.02) | 0.43(0.0003) | 0.30(0.01) | 0.37(0.0017) | 0.59(<.0001) |
| **G** | 68 | 0.37(0.002) | 0.26(0.03) | 0.34(0.004) | 0.25(0.04) | 0.29(0.01) | 0.48(<.0001) |
| **F** | 68 | 0.35(0.0035) | 0.20(0.09) | 0.30(0.01) | 0.25(0.04) | 0.25(0.04) | 0.45(0.0001) |

**S3b Table. Spearman correlations between RSV gene copy numbers and pro-inflammatory cytokines.**

|  |  |  | **Un-normalized** |  |  |
| --- | --- | --- | --- | --- | --- |
| **Un-normalized** | **N** | **Hu IL 1b** | **Hu IL 6** | **Hu IL 8** | **Hu TNF α** |
| **NS1** | 68 | 0.13(0.31) | 0.20(0.10) | 0.19(0.11) | 0.12(0.34) |
| **NS2** | 68 | 0.12(0.35) | 0.18(0.14) | 0.22(0.07) | 0.10(0.44) |
| **N** | 68 | 0.10(0.42) | 0.23(0.05) | 0.20(0.10) | 0.08 (0.52) |
| **G** | 68 | 0.13(0.28) | 0.25(0.04) | 0.22(0.07) | 0.13(0.30) |
| **F** | 68 | 0.12(0.33) | 0.19(0.13) | 0.18(0.14) | 0.14(0.27) |
|  |  |  |  |  |  |
|  |  |  |  |  |  |
|  | **Normalized** | | | | |
| **Normalized** | **N** | **Hu IL 1b** | **Hu IL 6** | **Hu IL 8** | **Hu TNF α** |
| **NS1** | 68 | 0.07(0.55) | 0.28(0.02) | 0.16(0.19) | 0.02(0.84) |
| **NS2** | 68 | 0.04(0.72) | 0.25(0.04) | 0.13(0.29) | -0.003(0.98) |
| **N** | 68 | 0.08(0.51) | 0.32(0.009) | 0.18(0.13) | 0.03(0.81) |
| **G** | 68 | 0.08(0.53) | 0.27(0.03) | 0.14(0.26) | 0.05(0.67) |
| **F** | 68 | 0.09(0.46) | 0.25(0.039) | 0.14(0.27) | 0.14(0.26) |

**S3c Table. Spearman correlations between RSV gene copy numbers and Th1 cytokines.**

| **Un-normalized** | | | | |
| --- | --- | --- | --- | --- |
| **Un-normalized** | **N** | **Hu IL 2** | **Hu IL 12 p70** | **Hu IFN g** |
| **NS1** | 68 | -0.03(0.83) | 0.23(0.06) | 0.23(0.06) |
| **NS2** | 68 | -0.02(0.86) | 0.19(0.11) | 0.14(0.25) |
| **N** | 68 | -0.06(0.61) | 0.22(0.08) | 0.21(0.10) |
| **G** | 68 | -0.07(0.56) | 0.21(0.08) | 0.16(0.19) |
| **F** | 68 | -0.05(0.71) | 0.22(0.07) | 0.19(0.13) |
|  |  |  |  |  |
|  | **Normalized** | | | |
| **Normalized** | **N** | **Hu IL 2** | **Hu IL 12 p70** | **Hu IFN g** |
| **NS1** | 68 | 0.20(0.10) | 0.34(0.005) | 0.43(0.0003) |
| **NS2** | 68 | 0.18(0.15) | 0.30(0.01) | 0.39(0.001) |
| **N** | 68 | 0.20(0.11) | 0.36(0.003) | 0.46(<.0001) |
| **G** | 68 | 0.17(0.17) | 0.30(0.01) | 0.37(0.002) |
| **F** | 68 | 0.10(0.41) | 0.26(0.03) | 0.35(0.004) |

**S3d Table. Spearman correlations between RSV gene copy numbers and Th2 cytokines.**

| **Un-normalized** | | | | | |
| --- | --- | --- | --- | --- | --- |
| **Un-normalized** | **N** | **Hu IL 4** | **Hu IL 5** | **Hu IL 9** | **Hu IL 13** |
| **NS1** | 68 | 0.19(0.12) | 0.14(0.25) | 0.20(0.10) | 0.05(0.67) |
| **NS2** | 68 | 0.14(0.25) | 0.16(0.20) | 0.16(0.18) | 0.02(0.86) |
| **N** | 68 | 0.15(0.21) | 0.15(0.22) | 0.20(0.10) | 0.07(0.58) |
| **G** | 68 | 0.16(0.21) | 0.10(0.40) | 0.17(0.17) | 0.04(0.74) |
| **F** | 68 | 0.12(0.31) | 0.18(0.13) | 0.23(0.06) | 0.08(0.54) |
|  |  |  |  |  |  |
|  |  |  |  |  |  |
|  | **Normalized** | | | | |
| **Normalized** | **N** | **Hu IL 4** | **Hu IL 5** | **Hu IL 9** | **Hu IL 13** |
| **NS1** | 68 | 0.28(0.02) | 0.46(<.0001) | 0.38(0.002) | 0.38(0.002) |
| **NS2** | 68 | 0.24(0.05) | 0.43(0.0003) | 0.35(0.004) | 0.35(0.004) |
| **N** | 68 | 0.28(0.02) | 0.50(<.0001) | 0.42(0.0003) | 0.42(0.0004) |
| **G** | 68 | 0.26(0.03) | 0.39(0.0009) | 0.33(0.006) | 0.31(0.01) |
| **F** | 68 | 0.20(0.09) | 0.40(0.0007) | 0.32(0.008) | 0.29(0.02) |

**S3e Table. Spearman correlations between RSV gene copy numbers and regulatory cytokines.**

| **Un-normalized** | | | | |
| --- | --- | --- | --- | --- |
| **Un-normalized** | **N** | **Hu IL 10** | **Hu IL 17** | **Hu IL 1ra** |
| **NS1** | 68 | 0.24(0.047) | 0.14(0.24) | 0.33(0.006) |
| **NS2** | 68 | 0.20(0.11) | 0.11(0.39) | 0.30(0.01) |
| **N** | 68 | 0.21(0.09) | 0.10(0.42) | 0.32(0.009) |
| **G** | 68 | 0.21(0.08) | 0.09(0.45) | 0.37(0.002) |
| **F** | 68 | 0.18(0.14) | 0.12(0.32) | 0.29(0.02) |
|  |  |  |  |  |
|  |  | **Normalized** | | |
| **Normalized** | **N** | **Hu IL 10** | **Hu IL 17** | **Hu IL 1ra** |
| **NS1** | 68 | 0.39(0.001) | 0.30(0.01) | 0.30(0.01) |
| **NS2** | 68 | 0.34(0.004) | 0.26(0.03) | 0.25(0.04) |
| **N** | 68 | 0.42(0.0004) | 0.30(0.01) | 0.30(0.01) |
| **G** | 68 | 0.35(0.004) | 0.25(0.04) | 0.29(0.02) |
| **F** | 68 | 0.33(0.006) | 0.25(0.04) | 0.25(0.04) |

**S3f Table. Spearman correlations between RSV gene copy numbers and maturational cytokines.**

| **Un-normalized** | | | | | | | |
| --- | --- | --- | --- | --- | --- | --- | --- |
| **Un-normalized** | **N** | **Hu IL 7** | **Hu IL 15** | **Hu PDGF bb** | **Hu FGF basic** | **Hu G CSF** | **Hu VEGF** |
| **NS1** | 68 | 0.22(0.07) | 0.10(0.42) | 0.16(0.19) | 0.34(0.005) | 0.03(0.79) | 0.11(0.38) |
| **NS2** | 68 | 0.26(0.03) | 0.068(0.58) | 0.16(0.19) | 0.24(0.04) | 0.06(0.60) | 0.13(0.29) |
| **N** | 68 | 0.23(0.06) | 0.07(0.59) | 0.19(0.12) | 0.28(0.02) | 0.06(0.62) | 0.12(0.33) |
| **G** | 68 | 0.25(0.04) | 0.08(0.54) | 0.13(0.29) | 0.30(0.01) | 0.07(0.58) | 0.12(0.32) |
| **F** | 68 | 0.24(0.05) | 0.09(0.44) | 0.17(0.17) | 0.26(0.03) | 0.06(0.65) | 0.09(0.48) |
|  |  |  |  |  |  |  |  |
|  |  |  |  |  |  |  |  |
|  | **Normalized** | | | | |  |  |
| **Normalized** | **N** | **Hu IL 7** | **Hu IL 15** | **Hu PDGF bb** | **Hu FGF basic** | **Hu G CSF** | **Hu VEGF** |
| **NS1** | 68 | 0.33(0.006) | 0.39(0.0009) | 0.41(0.0005) | 0.48(<.0001) | 0.23(0.05) | 0.22(0.07) |
| **NS2** | 68 | 0.29(0.02) | 0.36(0.003) | 0.38(0.002) | 0.43(0.0003) | 0.21(0.09) | 0.19(0.12) |
| **N** | 68 | 0.37(0.002) | 0.43(0.0003) | 0.45(0.0001) | 0.49(<.0001) | 0.27(0.02) | 0.26(0.03) |
| **G** | 68 | 0.30(0.01) | 0.34(0.004) | 0.35(0.003) | 0.44(0.0002) | 0.20(0.11) | 0.19(0.12) |
| **F** | 68 | 0.31(0.01) | 0.30(0.01) | 0.33(0.006) | 0.36(0.002) | 0.21(0.09) | 0.18(0.15) |

**S3g Table. Spearman correlations between RSV gene copy numbers and chemoattractant cytokines.**

| **Un-normalized** | | | | | | | | |
| --- | --- | --- | --- | --- | --- | --- | --- | --- |
| **Un-normalized** | **N** | **Hu Eotaxin** | **Hu GM CSF** | **Hu IP-10** | **Hu MCP 1 MCAF** | **Hu MIP 1a** | **Hu MIP 1b** | **Hu RANTES** |
| **NS1** | 68 | 0.04(0.73) | 0.12(0.35) | 0.53(<.0001) | 0.34(0.005) | 0.08(0.50) | 0.08(0.50) | 0.23(0.06) |
| **NS2** | 68 | 0.07(0.57) | 0.07(0.55) | 0.48(<.0001) | 0.31(0.01) | 0.11(0.37) | 0.07(0.55) | 0.19(0.13) |
| **N** | 68 | 0.05(0.68) | 0.08(0.53) | 0.54(<.0001) | 0.34(0.005) | 0.08(0.51) | 0.12(0.33) | 0.21(0.09) |
| **G** | 68 | 0.09(0.47) | 0.05(0.66) | 0.47(<.0001) | 0.33(0.006) | 0.13(0.31) | 0.12(0.32) | 0.20(0.11) |
| **F** | 68 | 0.03(0.78) | 0.12(0.34) | 0.47(<.0001) | 0.30(0.01) | 0.08(0.51) | 0.12(0.33) | 0.19(0.13) |
|  |  |  |  |  |  |  |  |  |
|  |  |  |  |  |  |  |  |  |
|  | **Normalized** | | | | | | | |
| **Normalized** | **N** | **Hu Eotaxin** | **Hu GM CSF** | **Hu IP-10** | **Hu MCP 1 MCAF** | **Hu MIP 1a** | **Hu MIP 1b** | **Hu RANTES** |
| **NS1** | 68 | 0.33(0.006) | 0.36(0.003) | 0.54(<.0001) | 0.15(0.23) | 0.23(0.06) | 0.31(0.009) | 0.45(0.0002) |
| **NS2** | 68 | 0.32(0.008) | 0.32(0.008) | 0.51(<.0001) | 0.14(0.26) | 0.20(0.11) | 0.28(0.02) | 0.41(0.0007) |
| **N** | 68 | 0.37(0.002) | 0.36(0.002) | 0.59(<.0001) | 0.16(0.18) | 0.25(0.04) | 0.34(0.005) | 0.47(<.0001) |
| **G** | 68 | 0.29(0.01) | 0.30(0.01) | 0.48(<.0001) | 0.18(0.15) | 0.24(0.05) | 0.32(0.008) | 0.41(0.0006) |
| **F** | 68 | 0.25(0.04) | 0.28(0.02) | 0.45(0.0001) | 0.22(0.07) | 0.21(0.09) | 0.27(0.03) | 0.37(0.0025) |
