# Supplementary material for: The interdependencies of viral load, the innate immune response, and clinical outcome in children presenting to the emergency department with respiratory syncytial virus-associated bronchiolitis
Source: PLoS One. 2017 Mar 7;12(3):e0172953. doi: 10.1371/journal.pone.0172953 (PMC5340370; doi:10.1371/journal.pone.0172953)
Supplement: S4 Table — (DOCX) [file pone.0172953.s004.docx]

**S4 Table. Spearman correlations between raw RSV gene copy numbers and biomarker activity levels.**

|  |  | **Un-normalized biomarkers** | | | |
| --- | --- | --- | --- | --- | --- |
| **Un-normalized copy numbers** | **N** | **NW LDH** | **NW Caspase** | **NW MMP7** | **NW MPO** |
| **NS1** | 79 | 0.20(0.08) | 0.46(<.0001) | 0.17(0.14) | 0.28(0.01) |
| **NS2** | 79 | 0.29(0.01) | 0.50(<.0001) | 0.24(0.04) | 0.37(0.001) |
| **N** | 79 | 0.27(0.02) | 0.53(<.0001) | 0.24(0.03) | 0.35(0.002) |
| **G** | 79 | 0.32(0.004) | 0.54(<.0001) | 0.31(0.005) | 0.36(0.001) |
| **F** | 79 | 0.18(0.12) | 0.42(0.0001) | 0.22(0.047) | 0.29 (0.01) |

Abbreviations: LDH = Lactate dehydrogenase; MPO = myeloperoxidase; MMP-7 = matrix metalloproteinase-7 (MMP-7).
