# Supplementary material for: The interdependencies of viral load, the innate immune response, and clinical outcome in children presenting to the emergency department with respiratory syncytial virus-associated bronchiolitis
Source: PLoS One. 2017 Mar 7;12(3):e0172953. doi: 10.1371/journal.pone.0172953 (PMC5340370; doi:10.1371/journal.pone.0172953)
Supplement: S5 Table — (DOCX) [file pone.0172953.s005.docx]

**S5 Table. Raw RSV gene copy numbers between non-hospitalized and hospitalized infants.**

| **Transcript copy numbers of RSV genes** | **Non-hospitalized** | | **Hospitalized** | |  |
| --- | --- | --- | --- | --- | --- |
| **Un-normalized** | N | Median (Q1-Q3) | N | Median (Q1-Q3) | P-value |
| **NS1** | 38 | 53.87 (1.70-168.97) | 41 | 19.80(2.09-73.08) | 0.05 |
| **NS2** | 38 | 12.34 (3.20-26.63) | 41 | 4.81(0.08-22.98) | 0.12 |
| **N** | 38 | 57.28 (18.03-114.63) | 41 | 19.84(1.98-119.53) | 0.18 |
| **G** | 38 | 88.21 (18.37-245.44) | 41 | 46.94(1.89-147.94) | 0.21 |
| **F** | 38 | 35.35 (0.79-95.18) | 41 | 16.13(1.06-61.38) | 0.51 |

Median RSV gene copy numbers in table = actual median/10^4^. Differences between groups were evaluated by Wilcoxon-Mann-Whitney tests. P value <0.05 was considered significant.
